# Summer Clothing

**Published:** 1893-06

**Authors:** 


### SUMMER CLOTHING.

Evidently flannel is rapidly displacing linen and cotton for summer underwear here in the north. The most comfortable garments for hot weather are not, as many assume, those which are the lightest. The first essential is the power to absorb moisture. Linen and cotton are notoriously poor absorbents, while wool stands high in this respect. Flannel garments absorb the perspiration readily and keep the surface of the body comparatively dry. Another thing, when wearing linen or cotton, the clothing soon becomes wet and clammy by contact with the perspiring skin, and if one enters a current of air there is rapid evaporation of moisture and the body becomes cooled much too suddenly, in consequence of which disease often results. Of course, the same condition of things follows if the temperature falls and it comes on colder. In our climate, where such changes are extremely common, woolen garments are the only safe ones.

